# Immune Responses to Cancer: Are They Potential Biomarkers of Prognosis?

**DOI:** 10.3389/fonc.2013.00107

**Published:** 2013-05-17

**Authors:** Theresa L. Whiteside

**Affiliations:** ^1^Department of Pathology, University of Pittsburgh School of Medicine, University of Pittsburgh Cancer InstitutePittsburgh, PA, USA; ^2^Department of Immunology, University of Pittsburgh School of Medicine, University of Pittsburgh Cancer InstitutePittsburgh, PA, USA; ^3^Department of Otolaryngology, University of Pittsburgh School of Medicine, University of Pittsburgh Cancer InstitutePittsburgh, PA, USA

**Keywords:** cancer, immune system, T-cell signature, B-cell signature, prognosis

## Abstract

Recent technical improvements in evaluations of immune cells *in situ* and immune monitoring of patients with cancer have provided a wealth of new data confirming that immune cells play a key role in human cancer progression. This, in turn, has revived the expectation that immune endpoints might serve as reliable biomarkers of outcome or response to therapy in cancer. The recent successes in linking the T-cell signature in human colorectal carcinoma (CRC) with prognosis have provided a strong motive for searching for additional immune biomarkers that could serve as intermediate endpoints of response to therapy and outcome in human cancers. A number of potentially promising immune biomarkers have emerged, but most remain to be validated. Among them, the B-cell signature, as exemplified by expression of the immunoglobulin G kappa chain (IGKC) in tumor-infiltrating lymphocytes (TIL), has been validated as a biomarker of response to adjuvant therapy and better survival in patients with breast carcinoma and several other types of human solid tumors. Additional immune endpoints are being currently tested as potentially promising biomarkers in cancer. In view of currently growing use of immune cancer therapies, the search for immune biomarkers of prognosis are critically important for identifying patients who would benefit the most from adjuvant immunotherapy.

## Introduction

It has been well established that the development of cancer is associated with alterations in numbers and functions of immune cells in the peripheral circulation and especially at the sites of tumor progression. More recently, a possibility has emerged that immune measures could serve as biomarkers or as surrogate endpoints of clinical responses. However, despite considerable progress made in immune monitoring methods, it has been difficult to correlate changes in the host immune profile associated with cancer progression or cancer therapy to clinical endpoints. This difficulty in establishing meaningful correlations of immune with clinical endpoints has been variously attributed to the complexity of interactions between the host immune system and the tumor, an inadequate quality of immune monitoring used to measure these interactions and/or a failure to measure those aspects of immune responses that are most relevant to cancer progression. Recent progress in our understanding of cellular and molecular pathways involved in immune responses to cancer has greatly facilitated the selection of the most relevant immune endpoints to evaluate. Also, impressive technological advances in methods allowing for multiplex profiling of immune phenotypes, definition of regulatory cell subsets, identification of critical signaling molecules, and recognition of biologically important targets, all have increased our ability to begin to discover potential immune biomarkers of disease-free or overall survival.

The need for robust biomarkers of outcome in cancer is especially great today. In the era of personalized medicine and the rapidly expanding availability as well as the use of biotherapies in cancer, a requirement for immunologic biomarkers of therapeutic efficacy is especially strong. To help fulfill this requirement, the recently published recommendations of the joint SITC/NCI/FDA Taskforce for the development and use of biomarkers in immunotherapy trials offer guidelines for how to establish, standardize, and evaluate immune assays, so that they meet a biomarker designation (Butterfield et al., 2011a).

The question of whether and how the host immune system influences cancer development has been debated for decades. While studies in animal models of cancer strongly support the role of anti-tumor immunity in cancer development, progression, and therapy, evidence from human clinical trials is not clear or straightforward. This may be in large part due to profoundly immunoinhibitory effects human tumors exert (Huang et al., 2008; Whiteside, 2008b), with the result that cancer patients at best mount weak anti-tumor immune responses which are ineffective in controlling cancer progression (Whiteside et al., 2011). Given that tumor-induced immune suppression exists, promotes tumor escape, and thus creates a major problem for cancer therapy (Whiteside et al., 2011), it might be possible to use immunosuppression as a measure of patients’ immune competence or more precisely, of a deficiency in immune competence specifically targeted to the tumor. Thus, one could measure: (a) the degree of tumor-induced immune suppression by identifying a decrease in or absence of an anti-tumor immune response or (b) the degree of recovery from immune suppression after successful therapy (i.e., normalization of defective anti-tumor immune responses). In the first instance, the immune evaluation takes place at the time of diagnosis and prior to any therapy; in the second, it takes place prior to, during and after therapy. Both these approaches have been used in clinical trials with the hope that intermediate biomarkers of immune suppression as well as biomarkers of therapy-induced recovery can be identified. Because of the complexity of host-tumor interactions and limited immune monitoring capabilities, both have been difficult to implement in practice. Nevertheless, current evidence suggests that in a sufficiently large cohort of cancer patients with similar demographic and clinicopathologic features, the disease-related or therapy-related immunological alterations can be detected and reliably measured. Further, in a limited number of cases, such immune alterations have been shown to correlate with clinical outcome suggesting that upon validation, they might serve as future biomarkers of prognosis or response to therapy.

While promising, these results are preliminary, and in most cases, validation of these potential immune biomarkers remains to be performed. Nevertheless, there is an expectation that in the near future, some of these immune biomarkers will be serving as reliable intermediate endpoints facilitating the management of patients with cancer and providing insight into the selection of most effective therapeutic strategies for these patients. The purpose of this communication is to describe a handful of most promising immunological assays that are currently being evaluated as intermediate biomarkers of cancer progression, regression, or recurrence.

## “Immune Score” in the Tumor Microenvironment

Human solid tumors are nearly always infiltrated by immune cells (Whiteside, 1993). While the composition and extent of these inflammatory infiltrates vary among tumors, they represent solid evidence that the host immune system is engaged in surveying tumor development. The tumor microenvironment is known to have a strong impact on immune cells, and the identity, phenotype, localization, and density of immune cells present in the tumor has long been considered by immunologists to be critically important for tumor progression (von Kleist et al., 1987). Immune cells infiltrating human solid tumors have been extensively studied and found to exhibit unique phenotypic and functional characteristics (Pages et al., 2010; Fridman et al., 2012). While the presence and functions of T cells in the tumor were the major concern in many earlier studies, more recent data emphasize the diversity in cellular composition of immune tumor infiltrates in various tumor types, with B cells, NK cells, M1 and M2 macrophages, granulocytes, or mast cells contributing substantially to the “immune signature” that uniquely characterizes each solid tumor.

## The T-Cell Signature

Typing of tumor-infiltrating T lymphocyte (TIL) cells by immuno-histochemistry (IHC) and microscopic enumeration of these cells have been initially utilized to establish correlations between CD3+CD8+ T-cell infiltrations and prognosis (Naito et al., 1998). These studies have indicated a potential significance of CD8+ T cells as predictors of risk (Sato et al., 2005). However, human TIL were found to be functionally impaired relative to peripheral blood T cells of patients or of normal donors (Frey and Monu, 2008; Whiteside, 2010) and, in some instances, TIL were shown to contribute to tumor progression (Whiteside, 2006). Most of these early *in situ* studies of TIL were small, retrospective, and probably biased due to the use of imperfect manual cell counts. Nevertheless, they have initiated an intense debate as to whether TIL were harbingers of good or poor prognosis. As Rosenberg’s group at the NCI and other investigators were successful in expanding TIL for adoptive therapy, it appeared that if these cells were functionally deficient *in situ*, they clearly re-gained the ability to eliminate tumor cells upon culture in the presence of IL-2 (Dudley et al., 2003). Slowly, the realization that the host-tumor interactions are critical for determining the fate of immune cells found in the tumor microenvironment prompted the re-assessment of the role TIL play in cancer progression. It was the report on TIL in colorectal cancer (CRC) published by Galon et al. (2006) in Science in 2006 that convincingly altered our perception of the prognostic significance of these T cells in cancer. Using modern techniques of systems biology and an objective scoring system based on image analysis, Galon et al.’s (2006) data showed that the type, density and location of immune cells within tumors predicted positive clinical outcome. Subsequently, Fridman’s group has demonstrated by immunostaining of hundreds of CRC specimens that a strong local immune reaction, including CD3+, CD8+, and memory CD45RO+ T cells, correlates with a favorable prognosis regardless of the local extent of the tumor or the regional lymph node involvement (Pages et al., 2005; Fridman et al., 2011). At the same time, independent reports from various other laboratories on the nature and cellular composition of immune infiltrates into human solid confirmed prognostic significance of the T-cell signature in cancer (Pages et al., 2005; Fridman et al., 2011). T-cell infiltrates emerged as the stronger independent prognostic factor than the conventional clinicopathological criteria such as tumor size, depth of infiltration, differentiation, or the nodal status (Galon et al., 2012). Based on these results, a proposal has been crafted for a routine evaluation of the tumor microenvironment for the density, location, phenotype, and function of T cells in order to define “an immune score” for each tumor as a part of the standard pathologic examination (Galon et al., 2012). While the proposal for the use of the immune score in pathology is supported by impressive globally collected data, it remains unclear whether and how soon this practice will be embraced by the pathologists. Of concern are issues related to the standardization of methods for a routine clinical use and requirements for automated image analyses. Nevertheless, at present, the immune score emerges as the first immunologic marker of risk in cancer with a potential to be incorporated into prognostically relevant immune classification of human CRC equal to or better than the conventional TNM classification (Galon et al., 2012).

## The Frequency of Tumor-Specific T Cells in the Circulation

In addition to scoring T cells at tumor sites, the frequency and functions of T cells circulating in the peripheral blood of cancer patients have been examined as potential biomarkers. The availability of standardized single-cell assays able to detect tumor-antigen-specific T cells (ELISPOT, cytokine flow cytometry (CFC), and tetramer binding) has facilitated evaluation of epitope-specific T cells as potential biomarkers (Britten et al., 2011). These assays, especially ELISPOT, have been standardized for serial monitoring (Britten et al., 2011) and can be reliably utilized to measure the frequency of epitope-specific T cells in blood or body fluids. While both CFC and tetramer assays require flow cytometry and thus are restricted to facilities equipped with a cytometer operated by a skilled technologist, ELISPOT is not. Perhaps for this reason, ELISPOT has become the most widely used single-cell monitoring assay. Also, while CFC and tetramer assays only measure the frequency of cells expressing a particular marker, ELISPOT defines the frequency of T cells able to respond to the recognized epitope by cytokine (e.g., IFN-γ) production. Thus, ELISPOT detects functional T cells (Whiteside, 2008a). In a recent ECOG-sponsored 1696 Phase II multi-center trial testing vaccination with melanoma peptides delivered alone, with GM-CSF, IFN-α2b, or both cytokines to HLA-A2+ patients with metastatic melanoma, we serially monitored the frequency of CD8+tetramer+ (tet+) T cells, their differentiation stages, and ELISPOT responses of CD8+ T cells to the vaccinating peptides (Schaefer et al., 2012). When these immunologic results were related to patients’ clinical responses, only IFN-γ ELISPOT results correlated with clinical responses, and neither the frequency of CD8+tet+ T cells in the periphery nor their differentiation stage were significant correlates of outcome (Schaefer et al., 2012). The data suggested that only the functional status of tumor peptide-specific CD8+ T cells, and not their phenotype or differentiation, served as a relevant biomarker for correlating immune and clinical responses to a peptide-based anti-tumor vaccine (Schaefer et al., 2012).

It has been suggested that the breadth of an immune response to tumor-associated peptides rather any one expanded response to a single peptide might correlate better with clinical response to immune therapy, especially to anti-tumor vaccines (Butterfield et al., 2003). In aggregate, data from multiple clinical studies suggest that patients who are able to mount immune responses to multiple as opposed to a single tumor-associated peptide have better prognosis. In the melanoma vaccine study discussed above, the breadth of immune responses (defined as an “immune score”) was greater for patients who remained disease-free than those with progressive disease (Schaefer et al., 2012).

## Apoptosis of CD8+ T Cells

Tumor-derived factors have been shown to induce death of immune cells at the tumor sites and in the peripheral circulation (Whiteside, 2010). The frequency of CD8+ T cells undergoing spontaneous apoptosis in the blood of patients with cancer was found to be significantly elevated relative to that in sex- or age-matched healthy controls (Hoffmann et al., 2002). CD8+ T cells were preferentially targeted for cell death compared to circulating CD4+ T cells (Tsukishiro et al., 2003). Further, evidence suggests that in cancer, tumor epitope-specific T cells are preferentially eliminated either directly via the Fas/FasL or Trail/TRAILR pathways (Albers et al., 2006) or indirectly through the release of tumor-derived exosomes (TEX) carrying death receptor ligands (Kim et al., 2005b). The propensity of T cells to undergo spontaneous apoptosis was measured by Annexin V binding and flow cytometry in several cohorts of patients with head and neck squamous cell carcinoma (HNSCC), and in these pre-clinical studies, the frequency of Annexin V-binding CD8+T cells was shown to discriminate patients with cancer from normal controls (Tsukishiro et al., 2003). However, using Annexin V binding, it was not possible to discriminate HNSCC patients with active disease from those with no evident disease following conventional therapies (Tsukishiro et al., 2003). The sensitivity of T CD8+ T cells to apoptosis was linked to high levels of Fas expression in the majority of these cells present in the peripheral circulation, and both the frequency of Fas+CD8+ T cells and expression levels of Fas on these cells were significantly increased in patients with advanced disease, a large tumor burden, and those with the metastatic lymph node involvement (Hoffmann et al., 2002; Tsukishiro et al., 2003). In more recent studies, Annexin V binding to CD8+ T cells in patients with HNSCC was found to be a less sensitive endpoint in discriminating patients from controls than the frequency of circulating CD8+ CCR7+ T cells, as discussed below (Czystowska et al., 2012). FasL+ microvesicles isolated from plasma of cancer patients have been linked to tumor progression, demonstrating that the presence of membrane-tethered FasL, and potentially of other molecules such as PDL-1 or TGF-β, could contribute to apoptosis of anti-tumor effector cells and thus to tumor escape (Whiteside, 2013). These studies suggest that the presence of death-inducing ligands or other immunosuppressive cargo in TEX might have prognostic value in patients with cancer (Whiteside, 2013).

## The Differentiation Status of CD8+ T Cells

Although the functional potential of tumor epitope-specific T cells *in situ* or in the peripheral circulation of patients with cancer has been shown in some studies to correlate with outcome (Kirkwood et al., 2009), performing of functional immune assays is demanding and costly. A search for alternative biomarkers suggested that T-cell differentiation, as measured by expression on CD8+ T cells of CCR7, a chemokine receptor for CCL19 and CCL21, discriminated cancer patients from normal controls (Czystowska et al., 2012).

We observed that in patients with HNSCC tested at the time of diagnosis, the frequency of CD8+CCR7+ T cells in the peripheral blood assessed by flow cytometry was significantly and often dramatically decreased relative to that in age- and sex-matched NC (Kim et al., 2005a). Using recursive partitioning statistics, we showed that the frequency of 28% CD8+CCR7+ T cells in the blood discriminated cancer patients from NC with a high degree of accuracy (Czystowska et al., 2012). We, therefore, inquired whether the paucity of CD8+CCR7+ T cells in the circulation at diagnosis and prior to any curative therapy could serve as a risk factor for the disease recurrence. Peripheral blood in a small cohort (*n* = 25) of previously untreated HNSCC patients with active disease was studied for the frequency of CD8+CCR7+ T cells at diagnosis. The patients were treated with conventional therapies and followed for up to 4 years for cancer recurrence. Remarkably, disease-free survival (DFS) was found to be significantly shorter for patients with fewer than 28% of circulating CD8+CCR7+ T cells at diagnosis compared to patients with >28% of these T cells (Czystowska et al., 2012). These results suggest that the frequency of CD8+CCR7+ T cells at diagnosis might play a role in cancer control regardless of definitive therapy these patients subsequently receive. Thus, a simple blood test at diagnosis could have a prognostic value in determining a possibility of the disease recurrence in HNSCC. These data are encouraging but preliminary, and the predictive value of this flow cytometry-based assay needs to be confirmed in additional much larger prospective studies. Nevertheless, the data provide preliminary evidence that immune biomarkers based on T-cell differentiation could be useful in predicting recurrence in HNSCC.

## The B-Cell Signature

To date, a search for promising immune correlates of cancer diagnosis, prognosis, and survival has been largely limited to T-cell responses. In their definition of the immune score, Fridman and colleagues rarely mention B cells or plasma cells. Yet, considerable evidence has existed for the presence of these cells in tumors, especially in breast cancer (Coronella et al., 2002). More recently, two independent reports have provided useful insights into the prognostic role of B cells in cancer. Schmidt et al. (2008, 2012) have reported convincing data that validate the B-cell signature as the most robust prognostic factor in breast cancer and other human tumors. These investigators identified the immunoglobulin G kappa chain (IGKC) as an immunologic biomarker of prognosis and response to chemotherapy in hundreds of patients with breast cancer, non-small lung cancer, and CRC (Schmidt et al., 2012). In this multi-institutional study, the IGKC was microscopically identified as a product of plasma cells present in the tumor stroma and was validated as a prognostic biomarker by the RNA- and protein-based expression studies independently performed in thousands of formalin-fixed, paraffin-embedded specimens at 20 different centers (Schmidt et al., 2012). Expression of the IGKC transcript was the strongest discriminator of patients with breast cancer with and without metastases among the 60 genes found in the B-cell metagene, while transcripts of the T-cell metagene had lesser prognostic significance (Schmidt et al., 2008). Infiltrates of both T and B cells were found to be associated with better prognosis (Schmidt et al., 2008). However, the most important finding was that IGKC predicted responses to neoadjuvant therapy in breast cancer and thus qualifies it as the first immune marker of response to cancer treatment. In view of an ongoing debate among tumor immunologists about the role of humoral vs. cellular immunity in tumor development, progression, and therapy, the above finding of the B-cell signature as a validated biomarker of prognosis and response to therapy provides a strong support for the role of humoral immunity in controlling cancer (Whiteside and Ferrone, 2012).

In support of this key role of the B-cell signature, Nielsen et al. (2012) recently reported that among TIL present in high-grade serous ovarian carcinomas, CD20+ B cells co-localized with activated CD8+ T cells and expressed markers of antigen presentation, including MHC class I, MHC class II, CD40, CD80, and CD86. These B cells were antigen experienced. The presence among TIL of both CD20+ B and CD8+ T cells correlated with increased patient survival compared with CD8+ T cells alone. Although these CD20+ B cells had an atypical CD27(−) memory B-cell phenotype, together with CD8+ T cells, they promoted favorable prognosis in ovarian cancer (Nielsen et al., 2012).

In an earlier study, Pretscher et al. (2009) have reported that in head and neck cancer (HNC), intra-tumoral CD20+ B cells were significantly more frequent in metastatic lesions than in primary tumors. Further, large numbers of peritumoral B cells together with increased numbers of intraepithelial CD8+ T cells in metastatic tumors were associated with favorable outcome in patients with oro-and hypopharyngeal carcinoma (Pretscher et al., 2009). The emerging evidence for a significant role of the B-cell signature as a biomarker of prognosis and possibly of metastasis in several human malignancies deserves careful attention particularly in view of novel insights into functional heterogeneity of this lymphocyte subset, which appears to play a pivotal role in regulating T-cell responses (Biragyn and Lee-Chang, 2012).

## Suppressor Cells in the Tumor Microenvironment

Accumulations of regulatory T cells (Treg) and myeloid-derived suppressor cells (MDSC) in human tumors and their increased frequency in the circulation of cancer patients have been widely reported (Marigo et al., 2008; Whiteside, 2012). Many reports, but not all, link these accumulations of CD4+FOXP3+CD25^high^ Treg to poor prognosis presumably due to suppression of anti-tumor responses by the accumulating Treg (Whiteside, 2012). In ovarian carcinoma, melanoma, breast cancer, and glioblastoma, the frequency of Treg among TIL correlated with tumor grade and reduced patient survival (Lanca and Silva-Santos, 2012). However, in other cancers, notably CRC, the presence and density of FOXP3+ Treg have been reported to predict favorable outcome and a better locoregional control of the tumor (Badoual et al., 2006; Salama et al., 2009). These discrepant results are based on the prevalent use of FOXP3 transcription factor expression as a marker of Treg. However, a recent comprehensive review of the prognostic significance of FOXP3+ T cells in 16 non-lymphoid cancers suggested that FOXP3 by itself is not a reliable marker of human Treg and that the tumor site, i.e., the tumor microenvironment, has a major impact on biologic effects of FOXP3+ Treg (deLeeuw et al., 2012; Whiteside, 2012). Because Treg are heterogeneous, consisting on many subsets of functionally distinct cells, and because no universal distinguishing marker for human Treg is currently available, their use as a biomarker of prognosis is limited and has to be taken with caution. Furthermore, current attempts to therapeutically deplete Treg might enhance tumor immunity in some patients but be detrimental in others (deLeeuw et al., 2012; Whiteside, 2012). Overall, the prognostic value of FOXP3+ Treg in cancer is questionable, although it is possible that the introduction of more specific assays for Treg might provide a more discriminating approach for evaluating their prognostic value.

A similar situation exists with respect to MDSC. Their accumulations in the tumor and blood of cancer patients have also been correlated to poor clinical outcome (Marigo et al., 2008; Raychaudhuri et al., 2011). The problem that confounds their use as biomarkers of outcome is twofold. First, their tremendous phenotypic and functional heterogeneity of the myeloid compartment creates a situation where everyone evaluates a different subset making it impossible to compare results. For example, a subset of HLA-DR^neg^Lin^neg^ MDSC present in the human peripheral blood contains cells with monocytic and granulocytic features, which can be subdivided into at least four distinct subsets (CD33+, CD11b+, CD15+, and CD14+) and which differ with respect to mechanisms used for suppression (Greten et al., 2011). A recently proposed immunophenotyping schema for MDSC, which utilizes multi-parameter flow cytometry, provides a unifying approach to future evaluations of the role these cells play in disease (Dumitru et al., 2012). This, however, does not eliminate the second problem of the sensitivity of CD15+ and CD33+ MDSC subsets to cryopreservation (Kotsakis et al., 2012). Only the frequency of CD14+ and CD11b+ subsets was not significantly decreased after PBMC cryopreservation, although their ability to produce ROS after *ex vivo* stimulation was lost (Kotsakis et al., 2012). These findings led to a conclusion that studies of human MDSC should be performed in fresh blood samples (Kotsakis et al., 2012). This requirement not only complicates monitoring of MDSC in clinical trials but significantly lessens their usefulness as potential prognostic biomarkers.

While both Treg and MDSC clearly play an important role in cancer progression and perhaps responses to immunotherapy, their usefulness as biomarkers of outcome or response to therapy has to await further development of monitoring assays that better reflect their biologic significance in cancer. In addition, a recent report on regulatory B cells (Biragyn and Lee-Chang, 2012) reminds us that this small subset of suppressor cells may have profound effects on the development of T-cell responses, further complicating the interpretation of anti-tumor immune suppression in disease.

## The Neutrophil-to-Lymphocyte Ratio

Chronic inflammation is closely associated with the development of certain human cancers. For example, inflammatory bowel disease predisposes to the development of CRC, and human papilloma virus (HPV) infection is associated with oropharyngeal squamous cell carcinoma. Evidence has accumulated that the total white blood count and especially the elevated neutrophil-to-lymphocyte ratio (NLR) measured prior to oncological therapies predicts adverse clinical outcome in patients with lung, breast, renal, ovarian, and HNC (Perisanidis et al., 2013). Further, the high NLR is a significant but not yet validated marker of poor response to chemotherapy (Perisanidis et al., 2013). These observations fit well with previously reported low lymphocyte counts in patients with cancer (Kuss et al., 2005). As discussed above, we have reported spontaneous apoptosis of circulating CD8+ antigen-responding effector T cells, leading to rapid lymphocyte turnover and depressed absolute numbers of T cell subsets in cancer patients tested prior to oncologic therapies (Whiteside, 2005). Together, these data identifying the high pretreatment NLR as a significant independent predictor of poor cancer-specific survival provide a strong rationale for considering a rapid validation of this promising biomarker.

## Cytokine Expression and Levels

Cytokine gene or protein profiling, whether by multiplex immunoassays, microarrays, or proteomics technologies, is especially well suited to evaluations of the tumor microenvironment. Given the key role it has in shaping local and systemic immune responses, events, and interactions between cells found in this milieu are of prime interest. Cytokines and chemokines mediate these interactions. Therefore, the potential for capturing polarization in the cytokine repertoire or differences in patterns of their production by immune or tumor cells and of relating them to a specific clinical response has a tremendous appeal. Systemic and local therapies with cytokines are becoming increasingly common, and there is a need for monitoring cytokine levels in relation to clinical endpoints. Such monitoring has greatly expanded our knowledge of the cytokine biology and has provided clinically useful information about cytokine involvement in human disease. In cancer, considered to be a Th2-dominant disease with excess of IL-4, IL-5, IL-10, and TGF-β production, a therapeutically driven shift back toward the Th1 profile is of interest, as it might correlate with immune and perhaps clinical recovery (Lucey et al., 1996). Indeed, plasma cytokines have been used as prognostic biomarkers in cancer (Hanash et al., 2008), with individual cytokines emerging as especially promising markers of survival. For example, elevated circulating levels of IL-6 have been associated with decreased survival in patients with cancer (Schafer and Brugge, 2007). The production of pro-inflammatory cytokines, IL-1β, IL-6, and TNFα, all of which facilitate tumor growth, in the tumor microenvironment might be due to STAT3 hyperactivation in both the tumor and immune cells (Yu et al., 2007). While cytokines and chemokines can be measured in the patients’ body fluids circulation by many different methods, multiplex bead immunoassays designed to work in conjunction with a Luminex-type instrument have all but replaced traditional ELISA. These assays allow for a simultaneous measurement of pro-inflammatory cytokines, Th1 vs. Th2-type cytokines, growth-promoting as opposed to suppressive cytokines, etc., in a small (0.5 mL) sample of a body fluid. The result is a quantitative profile of as many as 20–30 cytokines, and changes in this profile can be sequentially followed in the course of a clinical trial. Suppressed levels of Th1 cytokines (IFN-γ, IL-12, TNF-α) and elevated levels of Th2 cytokines (IL-4, IL-10, TGF-β) have been seen in patients with cancer, while the opposite pattern is seen in healthy individuals (Guida et al., 2007). A recent meta-analysis of cytokine profiles and their clinical significance has been published (Lippitz, 2013), and it summarizes the current insights into their diagnostic and prognostic value. To date, the use of cytokine/chemokine profiles as biomarkers of prognosis of cancer has been largely limited to retrospective analyses performed with banked serum specimens. As levels of these mediators in body fluids might be unstable upon extended cryopreservation (Butterfield et al., 2011b; Potter et al., 2012), it should be remembered that retrospective measurements of cytokines are highly prone to errors resulting from handling of samples, as also previously emphasized (Whiteside, 2002). To avoid pitfalls due to long-term storage and sample freezing/thawing, cytokine analyses should be performed with fresh, prospectively collected specimens tested in rigorously controlled assays (Butterfield et al., 2011b). However, even prospective monitoring for cytokines may be biased by the fact that current antibody-based assays measure soluble mediators, while those tethered to membranes of microvesicles present in body fluids remain undetected, as recently reported (Szczepanski et al., 2011). For this reason, the usefulness of cytokine/chemokine profiles as surrogate biomarkers of cancer progression remain limited and further studies linking these profiles with clinical outcomes are in order.

## Tumor-Derived Exosomes

Tumor-derived exosomes have recently come into the limelight as potential biomarkers in cancer. These membranous nanovesicles (50–100 nM in diameter) carry a large variety of cellular components, including proteins, RNA, microRNA, and DNA (Iero et al., 2008; Whiteside, 2013). TEX molecular content closely reflects that of tumor cells from which they originate, and thus TEX can serve as a sort of “liquid biopsy” in place of a conventional tissue biopsy. For this reason, molecular profiles of TEX are of great current interest. In our early studies of TEX, to establish a correlation of TEX with progression in HNSCC, we linked TEX functional activity (i.e., their ability to induce apoptosis of activated CD8+ T cells) with well-known markers of tumor progression, such as disease activity, lymph node involvement, and tumor stage (Kim et al., 2005b; Bergmann et al., 2009). The data showed that TEX present in plasma of HNSCC patients induced high levels of pan-caspase activity in CD8+ Jurkat cells or in activated primary CD8+ T cells (Kim et al., 2005b; Bergmann et al., 2009). This TEX-mediated effect was further enhanced when TEX were isolated from in patients with late-stage compared to early-stage tumors. More recent studies suggest that the total protein levels of TEX isolated from plasma and their molecular content reflect the tumor presence, its progression or regression after therapy and possibly its recurrence (Somasundaram and Herlyn, 2012). Exosome fractions obtained from the plasma of melanoma patients with stage IV disease had the highest protein concentrations relative to exosomes of patients with less advanced disease. Also, stage IV patients with protein-poor exosomal fractions had a significant survival advantage over those with protein-rich exosomes (Peinado et al., 2012). Our own preliminary data similarly show that the protein content of isolated TEX (in μg/mL plasma) in patients with advanced melanoma are higher than those in patients with early disease. In aggregate, these data suggest that the protein content of isolated TEX alone may be a biomarker of prognosis in cancer. In murine melanoma, TEX contributed to metastatic invasion by carrying messenger proteins that direct bone-marrow-derived cells toward a pro-metastatic phenotype (Peinado et al., 2012; Somasundaram and Herlyn, 2012). We recently showed that exosomes isolated from sera of AML patients at diagnosis inhibited functions of NK cells via membrane-tethered TGF-β and observed by using western blots that levels of this exosome-bound cytokine decreased during remission (Szczepanski et al., 2011). TEX carry and present membrane-bound enzymes, receptors, and cytokines to target cells, serving as message carriers and delivering signals to immune and tissue cells (Iero et al., 2008; Whiteside, 2013). Because membrane-bound proteins often have greater biological effects than their soluble counterparts, analysis of TEX might be of greater prognostic value than are measures of serum proteins. Currently, methods for capture of TEX to separate them from exosomes secreted by normal cells in the plasma of cancer patients are under development. With this technical improvement, the potential value of TEX as biomarkers of the tumor fate during and after therapy can be confirmed in future prospective studies.

## Conclusion

Despite considerable recent progress in the type and quality of immunologic monitoring applicable to clinical trials, linking results of correlative immunologic studies with clinical endpoints has been difficult and frustrating. In part, this might reflect the biologic variability inherent to the immune system and a demanding nature of serial immunologic studies, which require specialized and highly proficient laboratory support. It is also likely that the characteristics of tumor cells influence the potential of immune responses to serve as useful biomarkers of prognosis. Nevertheless, a number of promising immunologic biomarkers have emerged recently, perhaps as a result of improved high-through technologies incorporating proteomic and genomic platforms and of greater attention to changes in personalized immune profiles of cancer patients treated with conventional, biologic, or combination therapies. Most of these changes, whether reflecting cancer presence, progression, or response to therapy, have a potential to serve as intermediate biomarkers of outcome. However, only few, including T-cell and B-cell immune signatures or the NLR, have been validated in independent clinical studies encompassing large cohorts of patients with different tumor types (Schmidt et al., 2008, 2012; Perisanidis et al., 2013). Most of the other immune biomarkers showing promise in preliminary evaluations have yet to be validated, and much work remains to confirm their reliable association with clinical endpoints. Considering the data reviewed above, it would be reasonable to assume that good prognosis associates with CD8+ T cell and CD20+ B cell infiltrates into the tumor, while accumulations of Treg or MDSC are linked with bad outcome. Further, given the functional differences between immune cell subsets, a distinct outcome could be expected from Th1 vs. Th2 or vs. Th17+CD4+ cell profiles or from tumors infiltrated by M1 vs. M2 cells. Not only the phenotype, the frequency and functions of these various cell subsets might be important for prognosis but also their localization at the tumor site and their compartmentalization between the tumor and the periphery. Thus, substituting IHC by sophisticated but much more reliable image analysis coupled with precise clinicopathologic analysis and clinical follow-up in well-defined, relatively uniform cohorts of patients offers the best option for confirming immune endpoints as biomarkers of outcome. This approach has been most successfully used by the French investigators in CRC, and their experience in hierarchical clustering of signature T-cell transcripts (Galon et al., 2006; Camus et al., 2009; Pages et al., 2010; Fridman et al., 2011, 2012) paves the way for integrating immune cell signatures with prognosis in other tumor types. Much work remains to confirm the reliable association of immune biomarkers with clinical endpoints and to validate each of these potentially promising biomarker in a series of prospective clinical trials.

## Conflict of Interest Statement

The authors declare that the research was conducted in the absence of any commercial or financial relationships that could be construed as a potential conflict of interest.
